# Mississippi Valley Dental Association

**Published:** 1885-04

**Authors:** E. G. Betty


### MISSISSIPPI VALLEY DENTAL ASSOCIATION.

FORTY-FIRST ANNUAL MEETING AT CINCINNATI, MARCH 4, 5 AND 6, 1885.

REPORTED FOR THE INDEPENDENT PRACTITIONER BY E. G. BETTY, D. D. S.

The meeting was called to order at 10 o'clock A. M., with President Van Antwerp in the chair. After the transaction of routine business the following programme was offered for consideration:

I.—Progress of Dental Education.

II.—Recent Theories Concerning Caries of the Teeth.

III.—New Remedies for the Treatment of Oral and Dental Diseases.

IV.—Improvements in Prosthetic Dentistry.

V.—New Methods and Materials for Filling Teeth.

VI.—Incidents of Office Practice.

The first subject being announced for discussion, Dr. Otto Arnold, of Ironton, O., presented a paper, of which the following is a synopsis: There is no doubt in the minds of any of us that the profession is progressing in many ways, and that, too, very rapidly ever since the "turnkey" was discarded, and attention was directed to the conservation of the organs of mastication. The dental practitioner is now paying more attention to the materia medica and therapeutics of dentistry than formerly, yet not to that degree the progress in other branches of the art demands. Many of the symptoms of first dentition require urgent treatment at the hands of the dentist, and he should be qualified to diagnose his case, and prescribe for it on general medical principles.

The eruption of the *dens sapientiae* often causes disturbances of the general system that call for remedial agents, and it is the office of the dentist to be prepared to treat rationally and relieve the intense suffering that frequently accompanies these dental lesions.

It is gratifying to know that more attention is now being devoted to this branch of the art, and that the colleges are, by their system of education, preparing the dentist of the future to deal in an intelligent manner with such diseases and disturbances as have their origin in the mouth.

Dr. A. Berry, of Cincinnati, also read a paper upon the same subject. The progress being made in dental education is very gratifying; formerly the practitioner was not a man of liberal education, as there were no colleges, no journals, no societies; the dentist was an isolated being, did not confer with his neighbor dentist, nor have anything of a social feeling towards others also practicing. Any one wishing to become a dentist was obliged to go to a tutor for practical instruction, and had to rely on the medical profession for such knowledge as he might need in anatomy, physiology, materia medica, chemistry, etc. The elevation of the profession to its present level was a gigantic task, and was only accomplished by the associated efforts of the colleges, the journals, and the societies. The founders of the colleges builded well and wisely, and time has demonstrated the wisdom of their efforts. In those early days there were no dental depots, no instruments and appliances for sale, with the exception of the "key" and a few badly made forceps. The dental depots in all our large cities now furnish all that is needed by the most exacting dentist, and are often the means of teaching a great deal by the manufacture and exhibition of improved appliances.

To further the progress of education the terms of the colleges should be lengthened; as it is now, the five months allotted to study are shortened by holidays, etc., to eighteen weeks, the two courses required yielding but thirty-six weeks in which to prepare the student for practice, which is entirely inadequate. The essays, discussions and clinics at the society meetings, aid very much in spreading knowledge, and the twenty journals published are a great boon to the profession. The enactment of laws by the various State legislatures for the regulation of practice has wrought much good, by excluding the incompetents. There should be increased care in the editing of our journals, and the articles of contributors should be more condensed, and to the point.

Dr. R. J. Parre, of Cincinnati, then read parts of a paper bearing upon progress made in the diagnosis of constitutional diseases having their origin in dental lesions.

Dental lesions are often productive of constitutional disturbances, and though there is no lack of ability in our profession to cope with them, yet the dental practitioner needs to be awakened to the necessity of preparing himself to take hold of them with intelligence. The progress made in dentistry during the past twenty-five years has been very great, and is evidenced by the colleges that educate, and the new and improved methods perfected as experience dictates.

The difference that exists between the professions of medicine and dentistry is mainly due to the fact that we are educated exclusively for the practice of a specialty. The age of the "turnkey" has passed away, and the medical profession has, in a measure, come to depend upon dentists for assistance and consultation in such cases as involve a detailed knowledge of our departments. When the mouth is the seat of disease, poisonous and acrid secretions find their way into the stomach, disorder it, and entail a whole train of constitutional effects, more or less serious in their character. Loosening of the teeth is one of the effects of scurvy, and this disease gives rise to complications that often have the most deleterious effects upon the system.

As an illustration, I may mention a case in which blood poisoning was produced from the secretions incident upon the disease set up by the root of a dead tooth in the antrum. The root was removed, antiseptic dressings injected into the antrum, and the constitutional symptoms all disappeared.

The time has arrived when the two professions must extend courtesies to each other, and I may conclude with the statement that dentists should contribute freely to the medical journals.

FIRST DAY P. M. SESSION.

*Prof. J. Taft*—The subject of professional education is one upon which there is a great variety of opinion. Some wish to have it confined to small limits; others would have it branch out somewhat, while other extremists would have it embrace the whole field of human knowledge. That is too much, for in this day the disposition in all branches of investigation is for each to confine himself to one thing, and study it thoroughly. There was also much difference of opinion in the past, but the workers who founded our colleges were practically a unit, and included such men as Harris, Koecker, the Parmleys, and others, in the East, and Taylor, Melancthon Rogers, and others, in the West. These men were in advance of their profession in their day, and were they living now, they would still lead the van. The Baltimore College and the Ohio Dental College were the outgrowth of the opinions and energies of these men. To them also is due the credit of having organized the associations. The American Dental Association, from the first, took a high stand in this direction, and has since maintained it. (The professor then read extracts from the proceedings of the "College Faculty Association," held at Saratoga Springs last August, showing what action had been taken by the colleges to perfect and elevate the standard of requirement, and the action taken regarding a preliminary English education.) From 1840–45 there was a rapid increase in the numbers of those engaged in the practice of dentistry. A change is now taking place, and it is owing to the influence of professional journals and associations, making the profession take a higher place as a science and in the estimation of the public. The opinions as to what elements should enter into a dental education are very diverse, and have been much discussed. It is a matter of impossibility for any man to go over the whole field of literature, and equally so of science. Aside from this, general knowledge is necessary to better facilitate the practice of dentistry, so that it will profit a man to gain as much knowledge as possible upon any subject bearing in any way upon his specialty. He who has a large stock of general knowledge has a better fund to draw upon; his intellectual training is much better; he is intellectually more vigorous, and the better able to practice any branch of medical science. The more the man has, the more power does he possess, and his influence upon those about him will be the greater in proportion.

The man with strength is the man who tells; the man with the most money is the most powerful among those with whom he comes in contact; and so it is with him who has the greatest knowledge and best mental training. In our discussions of this subject we are apt to forget that men differ greatly in capacity. A man asks, "What shall I study?" It is best for him to study that for which he is best suited, as he will accomplish more good in that way. The man who is perfect in prosthetic dentistry is just as honorable as he who confines himself to surgery, or the treatment of disease.

*Dr. A. O. Rawls*—I did not come prepared to say anything on this subject, though I have my views upon it. I have watched the progress as indicated in the journals, colleges, etc., and am satisfied with it, as compared with that of other professions, and am proud to be a member of the dental profession. Not only have we progressed rapidly, but we have outstripped all other professions, and stand at the head upon all topics pertaining to special science, art, and literature. In the science of biology, who is ahead to-day? Is it not the dentist? Where did we get our splints to reset fractured jaws, our plastic operations, etc.? Did we get them from medicine? No! We are apt, in noting the standard of dental education, to consider the standing of dental colleges as all-sufficient for the position we occupy; but it is not so.

Prof. Taft represents one of the best colleges in the world, and he sends out students who, if they conducted themselves strictly as they are educated, could not live, for the reason that the great public is not intelligent enough to appreciate such a standard.

*Dr. H. J. McKellops*—I did not expect to say a word upon this subject when I came to the meeting of this, the oldest society in the world, and one of which every one should be proud to be a member. We must be a profession by ourselves, or not be any. I take great interest in dentistry, and in the education of dentists. Mere graduation does not fit a man to diagnose conditions of the mouth. I wish every man here would read the life of John Hunter. He was a man who investigated for himself, and by his studies discovered that which made him a dentist. Forty years ago Dr. Patrick was a diamond setter; what is he to-day? One of the most prominent and honored men in the profession. If we are going to be a profession, let us *be* a profession, and not hitch on to anything. We have competent professors, and we must educate ourselves. I love dentistry, and that is why I go from place to place visiting the societies. If I did not I would rather stay at home, mix up a little amalgam in a cup, and imagine that this was the agent that was to revolutionize the world. (Prolonged applause.) The revolution we want is in the *clinic* rooms. The man we want is he who goes into the clinic room and *demonstrates;* not he who writes unintelligible symbols on the blackboard. I wish I had the language to express all I think upon this subject, and carry conviction with it.

*Dr. F. W. Sage*—I am not opposed to a liberal education, but I do not favor a smattering of many things. We should stimulate the young men to study that which is best for them to know.

*Dr. McKellops*—I wish to God that I was one of the distinguished men of the dental profession. Dentists did not go across the water to bring professional knowledge here. We teach those men over there, and they anxiously look to this country for every flash of light that sparkles from our distinguished men. What are the journals there? They are an almost exact *fac simile* of our own. If one sits down to make a living only, he does not become enlightened; to become so he must read, study, and search into nature, to discover her truths.

*Dr. A. Berry*—I heartily agree with our distinguished visitor from St. Louis. I can hardly say visitor either, for he is one of the old, though not of the earliest members of this association. The young men should follow his advice, by reading and studying all that comes their way, particularly all that bears upon our profession.

*Prof. Taft*—No one has advocated the study of all science, but no one can object to a reasonable preliminary education. Some reference has been made to the colleges as being responsible for the status of the profession. Bear in mind that the colleges are restricted in the results they can obtain; up to a certain point they lack the support they should have; they have gone on as fast as they can be sustained and supported. When students come prepared with a good English education, *then* the profession will have accomplished a great advance. It is coming; the public requires it, and the other professions demand it of us.

*Dr. McKellops*—I cordially endorse Prof. Taft's remarks, but they do not strike the key-note. As things are now constituted it is impossible for a dental college to give any demonstrator a sufficient fee to command all his time, and this is where the colleges miss it. If men are thoroughly drilled they will not be incompetent; if one is drilled by a demonstrator capable of thorough teaching, when he goes out of the college he will have acquired that which is most necessary. The clinic is where the young men should be taught, and thoroughly taught.

*Dr. A. G. Rose*—I wish to say a word in behalf of the young man in dentistry. He is hounded down by everybody, and said to know nothing, whereas he *does* know something, can do pretty good dentistry, and is able to take care of himself.

*Dr. G. W. Smith*—I imagine the trouble is, that when we leave college we settle down and make no further advance, by lapsing in our studies. It would save the colleges much trouble if dentists would take more students into their offices, and teach them studious habits.

*Dr. R. J. Parre*—A fair literary education is necessary, and if any man without it enters the profession, it is the fault of his preceptor. There is a future before the profession, and a great change for the better not far off. We have reached that point where we should make better history.

*Dr. Jas. Leslie*—Over the water there is a total absence among the dentists of that desire, so common here, to be recognized as practitioners of a branch of medicine.

*Dr. Geo. W. Keely*—Dr. McKellops sympathizes very much with the demonstrators, and rightly. I doubt if there is a dental college in the United States whose teachers are able to live upon the salaries paid to them. I think a great point of progress in dental education to lie in the establishment of four great schools, one each in the North, East, South, and on the Pacific Slope. (Applause.) We could then concentrate our energies upon them, endow them handsomely, and so allow the corps of teachers to devote their entire time to preparing students for the thorough and scientific practice of their profession. To secure this, laws should be enacted and enforced, requiring students to prepare themselves in these colleges.

Adjourned till Thursday morning.

(TO BE CONTINUED.)

